# Early assessment of high-intensity focused ultrasound treatment of benign thyroid nodules by scintigraphic means

**DOI:** 10.1186/2050-5736-2-18

**Published:** 2014-09-30

**Authors:** Huedayi Korkusuz, Niklas Fehre, Michael Sennert, Christian Happel, Frank Grünwald

**Affiliations:** 1 Department of Nuclear Medicine, University Hospital Frankfurt, Theodor-Stern-Kai 7, 60590 Frankfurt am Main, Germany

**Keywords:** High-intensity focused ultrasound ablation, Thyroid nodule, Ablation techniques, Radionuclide imaging, ^99m^Tc-MIBI, ^99m^Tc-pertechnetate

## Abstract

**Background:**

High-intensity focused ultrasound (HIFU) allows to inflict intracorporal thermal lesions without penetrating the skin or damaging the surrounding tissue. This analysis intends to assess the magnitude of HIFU-induced ablations within benign thyroid nodules using scintigraphic imaging with ^99m^Tc.

**Methods:**

Ten cold, hot, or indifferent nodules were treated using multiple pulses of HIFU to induce temperatures of around 85°C within the ablation zone. Pre- and posttreatment, uptake values of ^99m^Tc-pertechnetate or ^99m^Tc-MIBI were recorded. The pre-post reduction of nodular uptake was evaluated to assess ablation magnitude.

**Results:**

Relative nodular uptake in relation to total thyroidal uptake decreased after one session of HIFU in all cases. Median ^99m^Tc-MIBI uptake reduction was 35.5% (ranging from 11% to 57%; *p* < 0.1), while ^99m^Tc-pertechnetate scintigraphy showed a median uptake reduction of 27% (range 10% to 44%; *p* < 0.1). No major complications were observed.

**Conclusions:**

HIFU appears to be safe and is an easy to perform means of thermal ablation. This study shows that HIFU treatment in thyroidal nodules can be evaluated by scintigraphic means shortly after the intervention. Due to small sample size, the exact magnitude of HIFU ablation efficiency in thyroidal nodules remains a value to be assessed in a larger study.

## Background

Thyroid surgeries are among the most common ones in western countries; the prevalence of thyroidal nodules (TNs) in the general population of a developed society is rather high (found in 4%–8% with the use of palpation, in 19%–67% using ultrasound (US) [1], in Germany, 21.9% of the working population have undetected TNs [2]). Especially for the treatment of benign TNs, risks [3] and costs of surgery seem rather high. Many patients that face only cosmetic or swallowing problems, minimal risks for the development of malignancy or slight autonomous nodular overproduction, for many years were given only the alternative of radioisotope therapy (RIT) in case of hot nodules. Over the last decades, different approaches have been developed, favoring not a removal, but only the local elimination of the abnormal tissue. Recent studies indicate that minimally invasive thermal ablation using microwave [4,5] or radio frequency [6-8] might provide an efficient method for achieving this goal. Yet, they maintain, just like local ablation via ethanol injection [9,10], an invasive characteristic; a disadvantage, ablation by high-intensity focused ultrasound (HIFU), does not necessarily share. HIFU is in use for several years for the treatment of prostate cancer [11], uterine fibroids [12], and benign breast lesions [13] and is currently under development for liver and kidney tumors [14]. So far, the application of HIFU on the thyroid is only reported concerning a preliminary experimental animal study [15,16], and one single case study on a human TN [17]. The feasibility of the method for treatment of majorly solid nodules has been shown in a study in which human thyroids were treated with HIFU 2 weeks before the scheduled thyroidectomy and then assessed histologically [18].

Thermal ablation utilizes the necrotizing effects on living human tissue of temperatures between 60°C and 100°C, as vaporization and carbonation effects start to occur above 100°C only [19]. The ablated tissue presents itself as a coagulation necrosis that over time will be disintegrated by immunology cells, resulting in lasting shrinkage of the nodule.

Like radio frequency or microwave ablation, HIFU treatment is conducted under US guidance. As with ultrasonography, the targeted tissue's functional destruction is neither confirmable nor quantifiable at a sufficient level; treatment success cannot be evaluated during the procedure and thus requires a postinterventional investigation. Scintigraphic imaging has been shown to be a promising means for the purpose of analyzing microwave ablated TNs [4]. This analysis intents to verify whether this is true for HIFU ablation as well, and if so, assess HIFU ablation magnitude using scintigraphic imaging.

## Methods

This analysis was an open label study. Patients over 18 years of age with at least one thyroid nodule with no signs of malignancy who met the protocol's eligibility criteria were recruited.

### Inclusion criteria

Benign TNs that require treatment for associated problems like thyrotoxicosis, neck pain, throat hoarseness, swallowing disorders, discomfort, and/or because of cosmetic concern. Patient either refuses surgical or RIT or shows major contraindications to these both treatments.

### Exclusion criteria

The exclusion criteria are the presence of a malignant nodule (diagnosed by an atypical finding in cold nodules in preliminary ^99m^Tc-MIBI scintigraphy confirmed by histological indication of follicular proliferation based on fine needle aspiration biopsy (FNAB)) or critical closeness of the nodule to sensible structures such as recurrent nerve, trachea, esophagus, or carotid artery.

Generally, HIFU treatment was presented as an alternative to microwave ablation. All ten patients (two males, eight females, age ranging from 36 to 80 years (median 55 years), each with one target benign TN (six cold ones, four hot or indifferent)) were included.

Written informed consent was obtained from all ten patients. They were informed of the known possible adverse effects of HIFU ablation (skin burn, pain, vocal cord palsy, tracheal and esophageal injuries, recurrence of the treated nodule). Written informed consent was also obtained concerning all diagnostic actions. The study was approved by the ethics commission of the University hospital Frankfurt.

### Evaluation

At baseline, all nodules were examined by US to determine their volume, precise location, echogenicity, blood perfusion (using US Doppler) and elasticity (using US elastography). According to the US Doppler, all nodes were graded either type 1 if no nodular blood flow was detectable, type 2 if it was minimal, and type 3 if significant blood flow was marked. They were furthermore classified to be of echogenicity type 1 if hypoechogenic, type 2 if isoechogenic, and type 3 if hyperechogenic. They were graded ES 1 (blue) if soft, ES 2 (blue-yellow) if more soft than solid, ES 3 (yellow-red) if more solid than soft, and ES 4 if predominantly solid (red) [20].

Additionally, ^99m^Tc scintigraphy was performed to provide information about nodular functional activity. Scintigraphic imaging was performed using 75 MBq ^99m^Tc-pertechnetate 20 min postadministration with a scintillation camera (Mediso Nucline® TH/22; Mediso, Budapest, Hungary; acquisition time, 300 s; matrix, 128 × 128 × 16, low-energy collimator).

Regarding cold nodule imaging, further investigations to exclude malignancy were conducted in all cases. This was achieved by additional FNAB cytology of the tissue in question with subsequent histological examination as well as a secondary scintigraphic imaging using ^99m^Tc-MIBI. Generally, the follow-up of cold nodules was also done with ^99m^Tc-MIBI scintigraphy (441 MBq) using the same scintillation camera as the pertechnetate scintigraphy, only with prolonged acquisition time (500 s) and different matrix (256 × 256 × 16). All scintigraphic imaging was done in compliance to DGN guideline. Postintervention, the nodules were also examined using US, US Doppler, and US elastography.

### Equipment

The HIFU device used for treatment in this study was the EchoPulse® (Theraclion SA, Malakoff, France), capable of US-guided extracorporeal HIFU treatment with contemporaneous cooling of the treated area. Its centerpiece is the probe, emitting both the treatment HIFU (3 MHz, piezo-electrically generating 125 W of maximal sound power) as well as the imaging US (7.5–12 MHz), that is placed in the midst of the former one, so that the treatment focal point is always displayed in the center of the US image. Both acoustic waves pass through a layer of cooling liquid held by a plastic balloon. The circulating liquid (10°C) is cooling the adjacent skin above the treatment area and thus prevents it from any damage due to heating from energy lost by the treatment echo on its way to the focal point and heat conducted from there. One of the many built-in safety provisions is a laser-based distance measurement between the robotic arm (holding the probe) and some reference point on the patient's skin. During treatment, distance changes of above 1 mm automatically lead to instant pausing of pulse emission to avoid inaccurate lesion placement due to patient movement. After such an interruption, repositioning of patient and/or probe is required before treatment can continue.

### Method and technique

The treatment itself is planned in about 10 to 20 sagittal and transversal section plane US images by target area as well as sensible structures (trachea, carotid, skin) being defined in each of them by the performing physician. The device creates security margins of 2 mm to the carotid, 5 mm to the skin, and at least 3 mm to the trachea, which cannot be infringed even when overlapping with the designated target area.

The focal point itself has the approximate form of a rice corn of 2 mm diameter and 9 mm length; the ablation area is a field of many such ellipsoid voxels “standing” side by side (Additional file 1). During treatment, each such voxel receives one echo pulse of 4-s duration followed by a cooling period of 20–40 s, during which the robotic arm repositions the probe to focus the next site. The usage of pulsed treatment also helps to prevent the formation of prefocal cavitations, which could lead to unwanted lesions in non-focal areas [21]. By default, all voxels that overlap with the target area and do not infringe any security margins are treated, but can be deselected manually.

One principle during HIFU treatment for this study was to maximize ablation zone volume within the targeted nodes. Accordingly, depth placement of the voxels was chosen to run through the nodule center, and proposed treatment sites were only excluded from execution if overlapping with the target area was marginal. Prior to the main ablation procedure, some test pulses were emitted to the nodule to adjust the echo intensity to just below the point where the so called heat bubbles [22] (visible in US and indicating sufficient heating to induce necrosis in thyroid tissue) emerge. Energy delivery per site ranged from 87.6 to 192.8 J. The duration of the procedure was 68 min on average and very dependent on the number of sites treated as well as frequency of significant patient movement and following repositioning.

Dependent on patient pain toleration levels, treatment was performed either under local anesthesia with Mecain or no anesthesia at all.

One day after the intervention, functional imaging was repeated with ^99m^Tc-pertechnetate for hot and indifferent and with ^99m^Tc-MIBI for cold nodules to assess treatment success. As the total uptake of ^99m^Tc-MIBI or ^99m^Tc-pertechnetate in a specific area of the thyroid depends on many factors of current individual metabolism, nodular uptake is only evaluated as the background is adjusted and in relation to total thyroidal uptake.

### Statistics

Statistical analysis was done with the free R statistical software (developed by the R Development Core Team). As normal distribution could not be assumed, the tests for significant changes of uptake reduction, echogenicity, perfusion, or elasticity of the nodules were performed with non-parametric methods (Wilcoxon signed rank test). Significance is defined as *p* < 0.1.

Due to small sample size and unknown underlying distributions, correlations are reported using Kendalls *τ* and tested against the null hypothesis of *τ* = 0.

## Results

**Figure 1:**
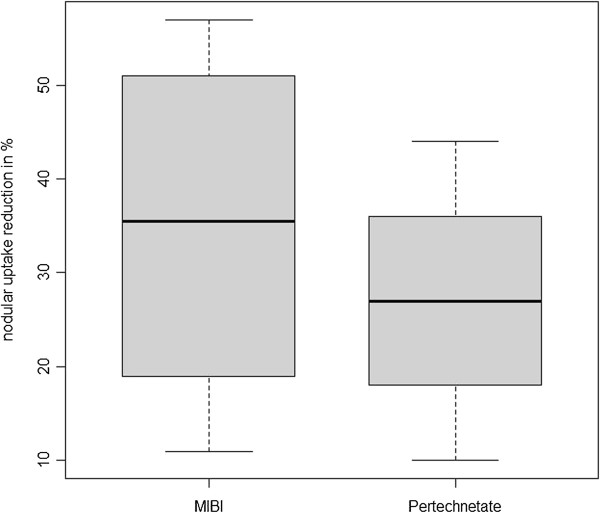
Relative nodular uptake reduction in percent of preablative nodular uptake.

**Figure 2:**
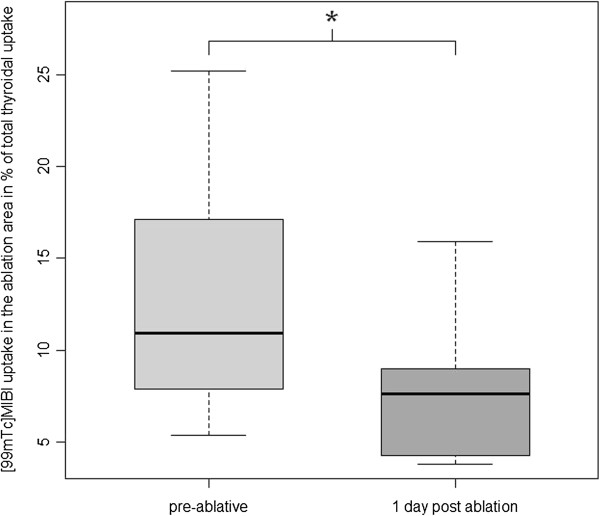
^**99m**^Tc-MIBI uptake reduction (*significant at *p* **= 0.016).**

**Figure 3:**
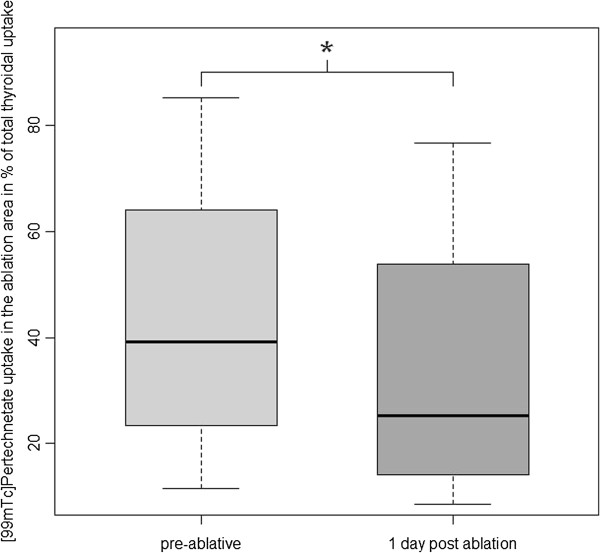
^**99m**^Tc-pertechnetate uptake reduction (*significant at *p* **= 0.063).**

**Figure 4:**
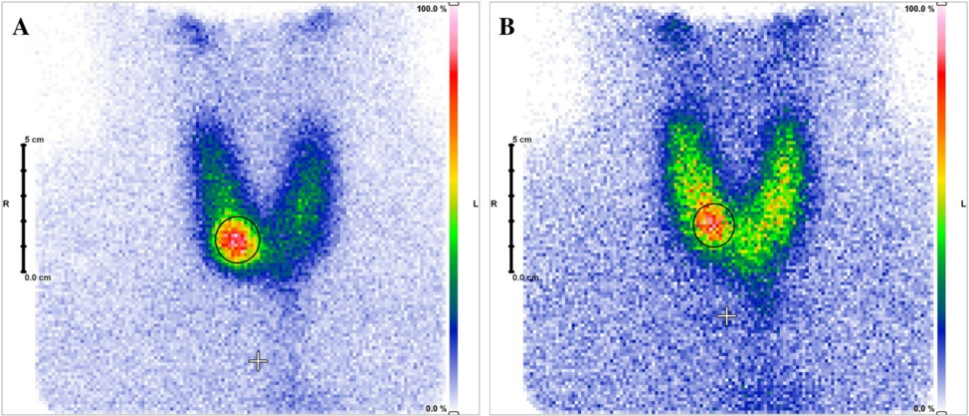
**Exemplary scintigraphical image.** Exemplary scintigraphic imaging of a hot nodule **(A)** pre- and **(B)** 1 day post-HIFU ablation. Nodular ^99m^Tc-pertechnetate uptake reduction as well as non-nodular uptake recovery due to ceased suppression by the focal autonomy (circle) is clearly visible.

Compared to the baseline, median nodular ^99m^Tc-MIBI uptake reduction was 35.5% (*p* = 0.016; ranging from 11% to 57%; see Figures 1 and 2), while ^99m^Tc-pertechnetate scintigraphy showed a median uptake reduction of 27% (*p* = 0.063; range 10% to 44%; see Figures 1, 3, 4) after the intervention.

**Figure 5:**
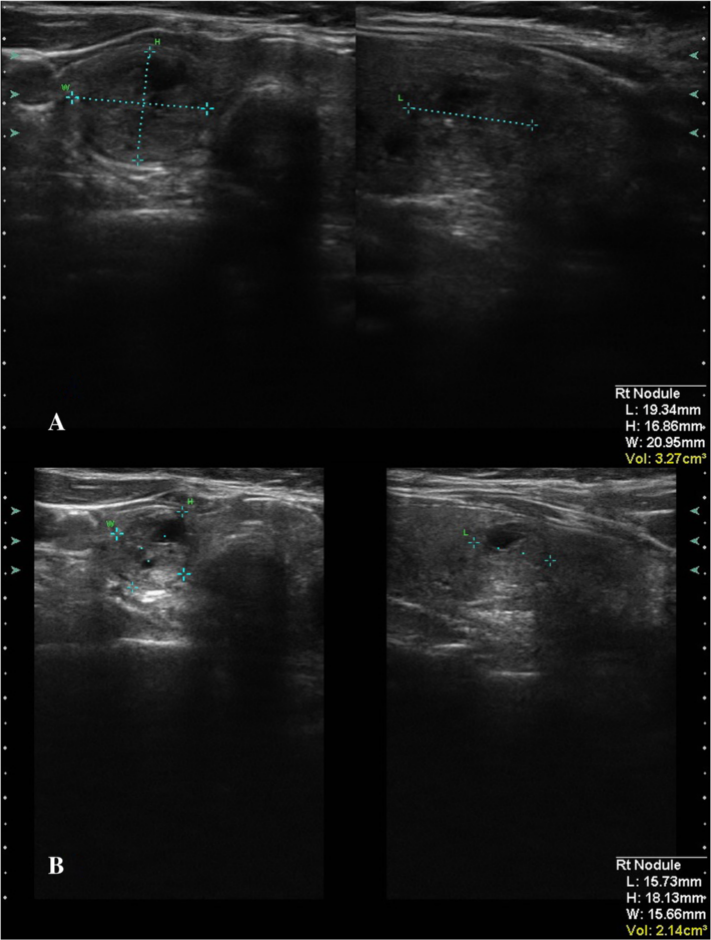
Plain US image of exemplary thyroidal nodule pre- (A) and 1 day postintervention (B).

**Figure 6:**
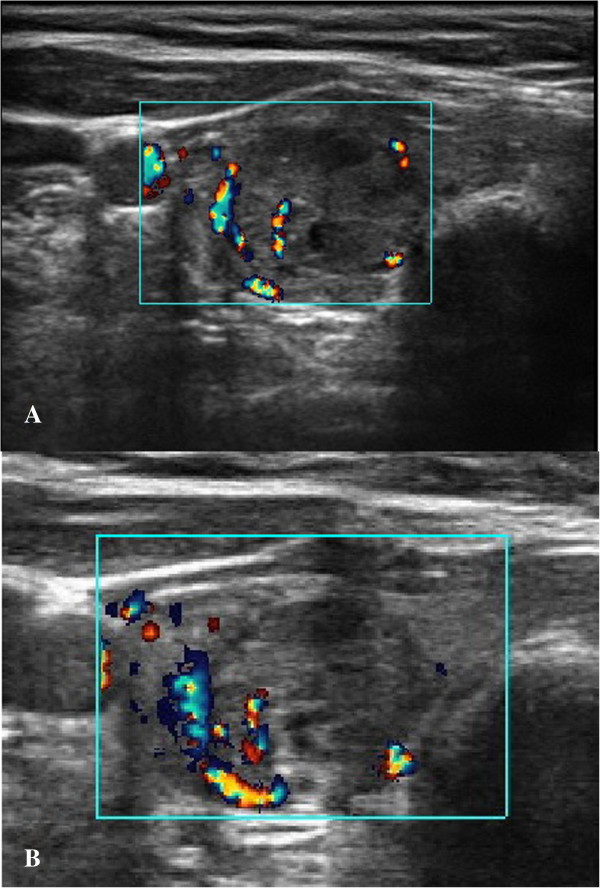
Doppler US image of exemplary thyroidal nodule pre- (A) and 1 day postintervention (B).

**Figure 7:**
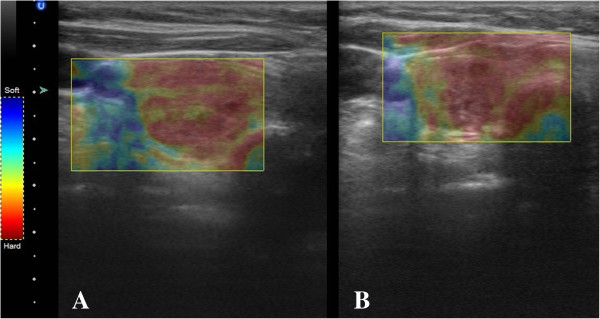
Echogenicity US image of exemplary thyroidal nodule pre- (A) and 1 day postintervention (B).

The US Doppler detected a decrease in nodular perfusion by one class in four cases (*p* = 0.036). Echogenicity decreased by one level in five cases (*p* = 0.019). Among them, three showed also a decrease in blood perfusion according to the US Doppler. An increased postablative nodular solidity by one ES score point was noticed in five cases (*p* = 0.019). Three of those showed no change in neither the US Doppler nor in the echogenicity score. (For exemplary pre-post images of US, US Doppler, and US-Elasticity, see Figures 5, 6, 7) respectively.)

The average treatment depth (distance from the skin to focal point) was 13.6 to 24.5 mm (median 19.85 mm). The total energy delivery (sum of all treatment pulses energy per patient) ranged from 5.65 to 12.46 kJ (median 8.4 kJ). The delivered energy per site (pulse energy each treatment voxel of a patient received) ranged from 87.6 to 192.8 J (median 157.9 J). The correlation between energy per site and either ^99m^Tc-MIBI uptake reduction or ^99m^Tc-pertechnetate uptake reduction was 0.46 (*p* = 0.28) and 0.33 (*p* = 0.75), respectively.

At baseline, the nodular volume ranged from 0.8 to 7.67 cm^3^ (median 3.185 cm^3^). *τ* correlations between nodular volume and ^99m^Tc-MIBI or ^99m^Tc-pertechnetate uptake reduction were −0.66 (*p* = 0.34) and −0.8 (*p* = 0.34), respectively.

Reported pain levels as well as treatment interruption frequency due to patient movement seemed to be independent of the dose of anesthetic or sedation given to the patient. Correlation between treatment duration and treated volume was 0 (*p* = 1).

## Discussion

The acceptance of thermal ablation of TNs as a viable alternative to surgery has increased during the last few years [23,24].

HIFU is in use for several applications (prostate, breast [25], uterus) and in testing for some others (tumors of liver [26] and kidney, primary [27] and secondary hyperparathyroidism [28], late-stage pancreatic tumors [29]).

This is the first study using HIFU to treat multiple patients with thyroidal nodes without a purely academic motivation (not just before thyroidectomy [18]), but trying to achieve a significant therapeutic benefit for the participating patients.

Generally, scintigraphic imaging of the thyroid allows the acquisition of functional information on a molecular level, with tracers being processed like their physiological metabolite counterparts. ^99m^Tc-pertechnetate is an iodine mimicry, and thyroidal uptake is correlating to hormone production activity. Nodules qualifying themselves as “hot” in ^99m^Tc-pertechnetate scintigraphy by an increased uptake of the radioisotope were viewed as benign, since malignancy of a hot nodule is very unlikely [30].

^99m^Tc-MIBI uptake levels depend mainly on mitochondrial activity and cellular membrane potentials; mitochondrial uptake is described to be significantly increased in malignant thyroid tissue [31].

^99m^Tc-MIBI scintigraphy is a method capable of excluding malignancy of hypofunctional TNs with high accuracy [32], of up to 92.2% [33]. Preinterventional rule out of malignancy on cold nodules was done by both ^99m^Tc-MIBI scintigraphy and FNAB, as the sufficiency for that task of ^99m^Tc-MIBI scintigraphy alone is still academically discussed [33]. The negative prediction value of combined ^99m^Tc-MIBI scintigraphy and FNAB is 96.3% [34].

Previous studies have shown that HIFU is capable of efficiently inducing cell death in the living pig muscle tissue, leading to non-perfusion and edema formation confirmed by MRI and the presence of histologically affirmed homogeneous necrosis in the targeted area [35]. Histological confirmation of local ablation effects of HIFU on the thyroid tissue has been reported both for ewe [15] and human [18] thyroid tissue. Therefore, the aim of this study was not to prove the destructive effects of HIFU on thyroid tissue in general but to evaluate how efficiently those effects in the non-surgical treatment of TNs can be detected and qualified using scintigraphy.

Postablative treatment assessment using Doppler, elastography, or plain US is fast, easy to perform, and relatively cheap. Yet, especially with the need to grade visual output, these methods are not very sensitive (none of them could detect any change in grade in more than 50% of the cases), and quantification of ablation magnitude remains vague. The inability of color-coded duplex sonography (Doppler) to reliably detect cold, hot, or indifferent TNs has been shown before [36]. Nodular perfusion decrease due to the ablation does not sufficiently change the nodular Doppler image to reasonably assess the treatment.

Assessment by elastography and plain US relies on changes in the nodules' physical characteristics that may or may not occur. Findings indicate that HIFU ablation can induce increased solidity and decreased echogenicity, but does not do so in an intensity that reliably changes corresponding scores.

The uptake of ^99m^Tc-MIBI as well as ^99m^Tc-pertechnetate on the other hand is dependent on the physiological functionality of the tissue in question and thus displays the attribute the conducting physician really is interested in, making functional imaging a better tool for ablation assessment. Treatment success can be measured in terms of relative nodular uptake reduction; ablation localization is visualized. Downsides of this method are higher costs and the patient's exposion to radiation. Additionally, the dependency between treatment coverage (how much of the nodular volume has been treated) and nodular uptake reduction is not a linear one, as thyroidal scintigraphy is a planar imagery, and thus, marker uptake of tissue ventral and dorsal of the nodular volume still is detected and accounted for as lying in the “region of interest”.

Operational shortcomings of the EchoPulse® device were the long treatment duration of 68 min on average (range of 42 to 96 min), mainly accredited to long cooling intervals and frequent readjustment pauses due to patient movement (correlation between treated volume and thus treated sites count and treatment duration was 0), as well as low imaging quality of the US system used for planning and controlling the procedure.

Technical constraints leading to the inability to treat the entirety of each nodule were the generously allocated security margins around sensible structures, the limitation to only one layer of treatment sites and only one direction of site alignment, as well as treatment depth confinement to approximately 3.5 cm.

Due to increased amounts of energy lost before reaching the focal point, target areas lying deeper within the body generally pose a problem of HIFU also described for other applications [29].

Despite of low significance, findings indicate that treatment success measured in relative nodular uptake reduction might be lower in larger nodules (correlation −0.66 to −0.8), most likely due to more nodular tissue lying ventral and dorsal of the treatment layer. Yet, this effect might be smaller than expected as with a theoretical intrafocal 85°C, especially not only pre- but also postfocal temperatures of above 60°C (injuring thyroidal tissue beyond repair [4]), seem likely. Such off focus lesions could also explain increased ^99m^Tc-MIBI and ^99m^Tc-pertechnetate uptake reductions at higher levels of energy delivery per site (correlation 0.33 to 0.46).

A recent study indicates that more degrees of probe rotation freedom may antagonize general main shortcomings of HIFU such as skin burn, long treatment time, and incomplete ablation [37].

In theory, the treatment of thyroidal nodes with HIFU could be undergone under general anesthesia in order to minimize pausing and slight targeting inaccuracies due to patient movement, but one major advantage of the method, its outpatient characteristic, would be lost.

Multiple treatments using HIFU on benign TNs might increase its effectiveness; as months after an ablation, nodular shrinkage might make parts of it accessible to HIFU treatment that were unreachable in previous sessions.

No major complications were observed. Subjective pain levels during pulse emission were moderate, but almost neglectable directly after treatment in most cases. One day postintervention, some patients developed slight erythrodermia and swelling in the treated region, presumably due to the consistent cooling during the ablation as well as a localized immunological reaction to the ablated tissue. Also, other authors have described HIFU to be a treatment method low on complications [38].

## Conclusion

HIFU treatment on benign TNs appears to be safe and is easy to perform. Scintigraphic imaging is a sensible means for postablative treatment assessment superior to US, Doppler, and US echogenicity. Therapeutic effectiveness of HIFU ablation on TNs is decent but could be enhanced further by increasing possible treatment coverage. Operational feasibility could be improved by decreasing treatment duration.

Due to small sample size, the findings of this study need to be verified by larger studies. Also long-term outcomes as well as the feasibility of multiple HIFU treatments on benign TNs should be investigated further.

## Abbreviations

FNAB: fine needle aspiration biopsy; HIFU: high-intensity focused ultrasound; RIT: radioiodine therapy; TN: thyroidal nodule; US: ultrasound.

## Competing interests

The authors declare that they have no competing interests.

## Authors’ contributions

KH, HC, and GF designed the study. KH carried out all HIFU treatments and collected US and scintigraphic data. FN and SM performed all statistical analysis. FN drafted the manuscript. KH, HC, and GF all revised the manuscript critically for important intellectual content. All authors read and approved the final version of the manuscript.

## Supplementary Material

Additional file 1 **Treatment site alignment model.** Visualization model of treatment site (voxels shown in red) alignment in a TN (blue). Treatment volume (sum of all voxels) is not congruent with nodular volume. Click here for file
