# Supplementary figures and images for: Early assessment of high-intensity focused ultrasound treatment of benign thyroid nodules by scintigraphic means
Source: J Ther Ultrasound. 2014 Sep 30;2:18. doi: 10.1186/2050-5736-2-18 (PMC4179864; doi:10.1186/2050-5736-2-18)

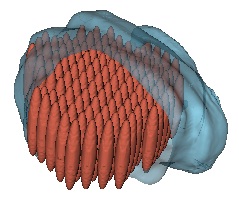

Supplement: Additional file 1 — Treatment site alignment model. Visualization model of treatment site (voxels shown in red) alignment in a TN (blue). Treatment volume (sum of all voxels) is not congruent with nodular volume. [file 2050-5736-2-18-S1.jpeg]
